# Outcome Assessment After Angioplasty With Catheter-Guided Thrombolysis and Stenting in Infrainguinal Peripheral Arterial Disease: A Prospective Observational Study

**DOI:** 10.7759/cureus.109705

**Published:** 2026-05-26

**Authors:** Kumar Kaushik, Jitendra Kushwaha, Shailendra Kumar, Saurabh Kumar, Ambrish Kumar, Amit Chaudhary, Saumya Singh

**Affiliations:** 1 Department of General Surgery, King George's Medical University, Lucknow, IND; 2 Department of Thoracic Surgery, King George's Medical University, Lucknow, IND; 3 Department of Radiodiagnosis, King George's Medical University, Lucknow, IND; 4 Department of Vascular Surgery, King George's Medical University, Lucknow, IND; 5 Department of Cardiothoracic and Vascular Surgery, King George's Medical University, Lucknow, IND; 6 Department of Surgery, King George's Medical University, Lucknow, IND

**Keywords:** ankle-brachial index (abi), critical limb-threatening ischemia, intermittent claudication, peripheral angioplasty and stenting, peripheral artery disease (pad)

## Abstract

Background and aim

Peripheral arterial disease (PAD) of the lower limbs causes significant morbidity and risk of limb loss. Endovascular interventions such as angioplasty with stenting may improve outcomes, but comparative prospective data are limited. This study aimed to evaluate the benefit of endovascular intervention (angioplasty with thrombolysis and stenting) and medical management in critical limb ischemia.

Materials and methods

A prospective observational study was conducted in a tertiary center. Forty-two adult patients with infrainguinal PAD were prospectively enrolled during the study period and received either medical management (Group A; n = 22) or endovascular intervention involving angioplasty with catheter-guided thrombolysis and nitinol stenting (Group B; n = 20). Outcomes, including pain relief, wound healing, need for amputation, mortality, and quality of life (measured by SF-36 and EQ-5D), were assessed over six months.

Results

Pain relief was observed in 72.7% of patients in Group A vs. 85% of patients in Group B. Wound healing showed a significant advantage in the intervention group: among those who presented with wounds, 81.8% healed in Group B vs. 20% in Group A (p = 0.0046). Amputation rates were 18.2% in Group A vs. 5% in Group B, and mortality was 18.2% in Group A vs. 5% in Group B, although these differences were not statistically significant.

Conclusions

Angioplasty with thrombolysis and stenting in patients with infrainguinal PAD yielded significant improvement in wound healing and self-care, with trends toward lower amputation and mortality compared with medical management.

## Introduction

Peripheral arterial disease (PAD) is defined as a chronic atherosclerotic occlusive disorder impacting the lower limbs. The burden of PAD is increasing worldwide due to an aging demographic as well as a growing incidence of risk factors, including hypertension, smoking, diabetes, and dyslipidemia. According to current epidemiological studies, more than 200 million cases of PAD exist worldwide [1]. The most significant risk factor is age, as the prevalence of disease rises sharply after the sixth or eighth decade [2]. Other associated factors include smoking, hypertension, diabetes, obesity, dyslipidemia, and chronic inflammatory conditions.

Anatomically, lower extremity arterial disease is divided into three segments: tibiopedal, femoropopliteal (FP), and aortoiliac (AI). Diehm et al. found that tibio-pedal disease was linked to advanced age and diabetes, while AI disease was linked to smoking, younger age, and male sex [3]. The TASC II classification primarily categorizes lesions into two main groups: AI lesions and FP lesions. There are four classifications, A through D, each ranging from occlusion to complete stenosis, encompassing both long and short segments, as well as bilateral and unilateral presentations [4].

Individuals affected with PAD can be classified as asymptomatic, intermittent claudication, atypical leg symptoms, or critical limb-threatening ischemia. Patients with asymptomatic peripheral artery disease exhibit a 5.6-fold increased risk of mortality from coronary artery disease as well as a 2.7-fold elevated risk of overall mortality. Reduced ankle-brachial index (ABI) and elevated ABI (>1.4), consistent with vessel calcification, have a very strong association with mortality and cardiovascular risk [5].

In intermittent claudication, patients complain of leg pain induced during exercise and relieved during the resting period. Patients most commonly present with pain in the calf; however, it can affect proximal muscles such as the buttocks, thigh, and hip. The severity of the disease is determined by the initial claudication distance. The most frequently affected infrainguinal area in individuals with intermittent claudication is the FP segment (FPS). Significant reduction in flow is caused when 50-75% of the diameter of the lumen of the FPS is compromised [6].

Acute limb ischemia is characterized by an abrupt reduction in arterial perfusion and symptoms that appear within two weeks [7]. Factors associated with the development of ALI include prior lower extremity revascularization, ABI < 0.6, and atrial fibrillation. It has a significant risk for major amputation (10-15%) and one-year mortality in the range of 15-40% [8].

Critical limb-threatening ischemia is clinically defined as an advanced stage of PAD when there has been tissue necrosis (gangrene or nonhealing ulcer) or foot pain at rest for at least two weeks. Absolute ankle pressures of <40-50 mm Hg and toe pressures of <30 mm Hg are typical values associated with ischemic rest pain [9].

PAD is commonly categorized using Rutherford and Fontaine classifications based on gangrene, ulcers, and clinical symptoms. As per the Rutherford classification, Stage I is asymptomatic, Stage IIa indicates mild claudication, Stage IIb indicates moderate to severe claudication, Stage III indicates ischemic rest pain, and Stage IV patients have ulceration or gangrene [10]. The Fontaine classification categorizes patients into Category 0, who are asymptomatic; Category 1, who have mild claudication; Category 2, having moderate claudication; Category 3, with severe claudication; Category 4, having ischemic rest pain; Category 5, having minor tissue loss; and Category 6, having major tissue loss [11].

The 2016 American College of Cardiology (ACC)/American Heart Association (AHA) guidelines stipulate that targeted ABI testing should be conducted in asymptomatic patients at heightened risk for PAD, defined as those over 65 years of age, those aged 50-64 years with a family history of PAD or atherosclerotic risk factors, individuals with diabetes accompanied by an additional risk factor, or those with established atherosclerosis in another vascular region (including coronary or cerebral) [12].

In the treatment of individuals with PAD, quitting smoking is the most crucial step. Patients with IC who quit smoking rarely develop chronic limb-threatening ischemia (CLTI) [13]. Supervised exercise is advised as a Class IA intervention for intermittent claudication according to the latest AHA/ACC guidelines [14]. Antiplatelet agents are classified as Class IA recommendations for individuals with symptomatic PAD according to the AHA 2016 Guideline Statement and the Society for Vascular Surgery Practice Guideline Statement [15]. Only two drugs that the FDA has approved so far to treat intermittent claudication are pentoxifylline and cilostazol.

Decisions regarding revascularization (endovascular vs. surgical) can be aided by anatomic classification of lesions using the Trans-Atlantic Inter-Society Consensus (TASC) classification and the Global Limb Anatomic Staging System (GLASS). While TASC C is in a gray area and should be surgically treated in more severe cases, TASC A and B lesions can be treated endovascularly, whereas TASC D lesions can only be treated surgically [16].

## Materials and methods

Study design and population

A prospective observational study was conducted at King George’s Medical University, Lucknow, from March 2024 to January 2025. The study observed 42 consenting adults (≥18 years) with chronic limb ischemia due to infrainguinal vessel disease classified as Fontaine III-IV or Rutherford IV-VI. Exclusion criteria included acute limb ischemia, proximal lesions, or refusal to participate. Patients who received medical management were kept in Group A, and those who were treated by angioplasty and stenting were kept in Group B.

Patients in Group A (n = 22) underwent lifestyle modification and received medical management. They were advised to quit smoking (if they were smokers) and maintain diabetes control for patients with type 2 diabetes mellitus. Patients were treated with a combination of medicines, including aspirin, cilostazol, rivaroxaban, and pentoxifylline. They were advised to exercise (at least four days a week), consume a balanced diet, and maintain adequate sleep.

**Figure 1:**
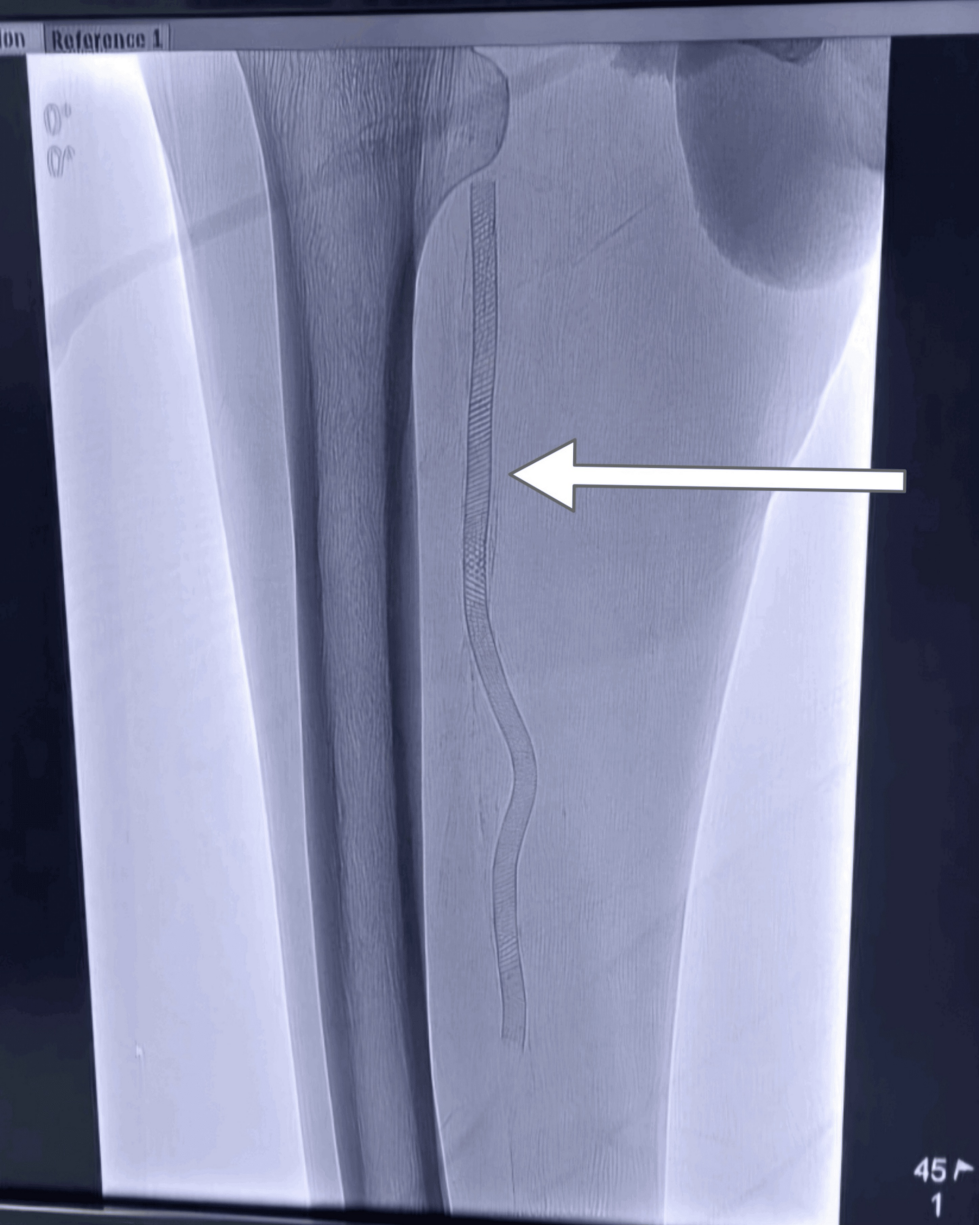
Stent placed across the affected segment The arrow shows the nitinol stent.

Patients in Group B (n = 20) underwent angioplasty with catheter-guided thrombolysis and implantation of a self-expanding nitinol stent, followed by standard postprocedural care. Patients were admitted to the interventional radiology ward. Patients were taken to the procedure room, local anesthesia was administered, and a 5-6 Fr sheath was inserted into the common femoral artery, most often by antegrade access. To assess the location and extent of the lesion, preliminary angiography was obtained. After that, a guidewire was inserted through the sheath into the occluded artery and passed beyond the lesion. A balloon was then instilled into the occluded artery, placed across the whole length of the lesion, and inflated. Angiography was then performed to check the patency of the occluded artery. A Supera self-expanding nitinol stent (Abbott Vascular Inc., Santa Clara, CA, USA) was then placed across the lesion segment, as shown in Figure 1, and angiography was performed to confirm blood flow across the previously occluded artery. The patient was then managed with heparin infusion from the sheath at the rate of 500 IU/hour for three days. After three days, the sheath was removed, and the patient was usually discharged from the hospital with advice for follow-up.

Assessments and outcomes

Demographic and comorbidity data were collected at baseline. Primary outcomes included pain relief, wound healing, limb amputation, and mortality at six months. Secondary outcomes included quality of life measured using the SF-36 questionnaire [17] and the EQ-5D questionnaire [18]. Follow-ups occurred on postoperative days 1, 3, and 7 and at one and six months.

Statistical analysis

Group comparisons involved t-tests for continuous variables and chi-squared tests for categorical variables. Statistical significance was set at p < 0.05.

## Results

Patient characteristics

Mean age was 51.7 years in Group A vs. 48.4 years in Group B. Male predominance was higher in Group B (90%) than in Group A (64%). Baseline comorbidities, ABI, BMI, and disease severity were comparable between groups.

Pain

In Group A, 72.73% of patients experienced pain relief compared to 85% in Group B. Conversely, 27.27% in Group A and 15% in Group B reported no pain relief. Although Group B had a higher proportion of patients reporting relief, the difference between the two groups was not statistically significant (chi-square = 0.35, p = 0.554), indicating that both treatments were similarly effective in providing pain relief (Table 1).

**Table 1:** Pain relief Relief from pain data were collected from patients in both groups. Data are presented as numbers (n) and percentages (%) within Groups A and B. Results were compared using the chi-square test, and p < 0.05 was considered significant.

Pain relief	Group A, n (%)	Group B, n (%)	Chi-square	p-value
Yes	16 (72.73)	17 (85)	0.35	0.554
No	6 (27.27)	3 (15)		

Wound healing

A total of 10 patients in Group A and 11 patients in Group B presented with wounds. Among these, wound healing was observed in two patients (20%) in Group A and nine patients (81.81%) in Group B. The chi-square test for the proportion of patients with wound healing between groups yielded a value of 8.02 with a p-value of 0.0046, indicating a statistically significant difference (Table 2).

**Table 2:** Wound healing Wound healing was compared among patients who presented with wounds. Data are presented as numbers (n) and percentages (%) within Groups A and B. Results were compared using the chi-square test, and p < 0.05 was considered significant.

Wound healing	Group A, n (%)	Group B, n (%)	Chi-square	p-value
Healing of wound	2 (20)	9 (81.81)	8.02	0.0046
No healing	8 (80)	2 (18.18)		

Amputation

In Group A, 18.18% of patients required amputation, while in Group B, only 5% required amputation. Although the proportion of amputations was lower in Group B, suggesting a possible clinical benefit of the intervention (angioplasty with thrombolysis and stenting), the difference was not statistically significant (chi-square = 0.61, p = 0.401) (Table 3).

**Table 3:** Amputation required in the patients Data are presented as numbers (n) and percentages (%) within Groups A and B. Results were compared using the chi-square test, and p < 0.05 was considered significant.

Amputation needed	Group A, n (%)	Group B, n (%)	Chi-square	p-value
Yes	4 (18.18)	1 (5)	0.61	0.401
No	18 (81.82)	19 (95)		

Mortality

In Group A, four patients (18.18%) died during the study period, compared to only one patient (5%) in Group B. Although mortality was lower in the group that underwent angioplasty with catheter-guided thrombolysis and stenting, the difference was not statistically significant (chi-square = 0.61, p = 0.434) (Table 4).

**Table 4:** Mortality Mortality during the study period. Data are presented as numbers (n) and percentages (%) within Groups A and B. Results were compared using the chi-square test, and p < 0.05 was considered significant.

Mortality	Group A, n (%)	Group B, n (%)	Chi-square	p-value
Yes	4 (18.18)	1 (5)	0.61	0.434
No	18 (81.82)	19 (95)		

Quality of life

**Figure 2:**
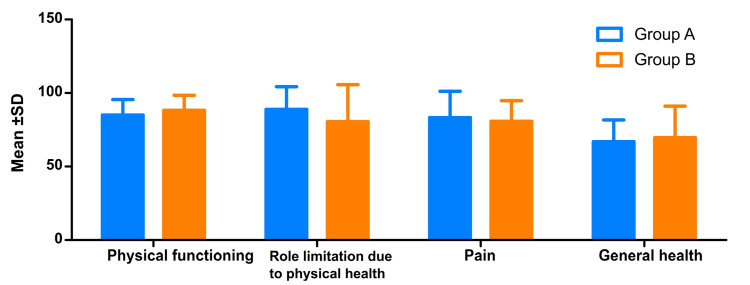
Physical health component Physical functioning, role limitation due to physical health, pain, and general health were compared. Data are presented in the bar graph as mean ± SD.

The mean physical functioning score was slightly higher in Group B (88.33 ± 10.15) than in Group A (85.00 ± 10.57), but the difference was not statistically significant (p = 0.341). Similarly, there was no significant difference in role limitation due to physical health, with scores of 88.89 ± 15.39 in Group A and 80.56 ± 25.08 in Group B (p = 0.238). The pain scores (83.33 ± 17.82 vs. 80.83 ± 13.96) and general health scores (66.94 ± 14.77 vs. 69.72 ± 21.38) also showed no significant variation (p = 0.642 and p = 0.653, respectively) (Figure 2).

EQ-5D questionnaire

In the mobility domain, a higher proportion of participants in Group B (73.68%) reported no problems compared to Group A (61.11%), although this difference was not statistically significant (p = 0.678). A statistically significant difference was observed in the domain of looking after myself, where all participants in Group B (100%) reported no problems, in contrast to only 61.11% in Group A (p = 0.011), indicating better self-care ability in Group B.

In the doing usual activities domain, the majority in both groups reported no issues (Group A: 88.89%, Group B: 84.21%), with no significant difference (p = 1.000). Regarding pain or discomfort, more participants in Group B (57.89%) reported no problems compared to Group A (27.78%), but the difference was not statistically significant (p = 0.153). Similarly, in the domain of feeling worried/sad or unhappy, a higher percentage of participants in Group B (78.95%) reported no problems compared to Group A (72.22%), although this was not statistically significant (p = 0.323).

Overall, while most domains did not show statistically significant differences, the domain of self-care (“Looking after myself”) demonstrated a notable improvement in Group B compared to Group A.

## Discussion

The basic purpose of this study was to enhance pain relief, wound healing, prevention of amputation, and quality of life by assessing the best possible treatment. When wound healing was compared among patients who presented with wounds at the time of inclusion, it was observed that nine (81.81%) of the 11 patients who presented with lower limb wounds showed wound healing in Group B compared with only two (20%) patients in Group A, indicating a statistically significant difference (p = 0.0046). This observation was found to be consistent with Armstrong et al. (2017), who evaluated wound healing and limb salvage rates after angiosome-targeted balloon angioplasty in critical limb ischemia (CLI), suggesting that the complete wound healing rate is better when the target foot lesion receives direct perfusion [19]. Thus, angioplasty with stent placement increases the perfusion of the affected part of the limb, leading to better wound healing.

Out of 10 patients who presented with wounds in Group A, four (40%) needed amputation due to worsening of the preexisting wound. However, the amputation requirement result in Group B was significantly better, in which only one (9.09%) patient needed amputation. The findings of this study were consistent with the Armstrong et al. study, which utilized data from January 2006 to December 2014. Conservative therapy was associated with significantly higher odds of major amputation or inpatient death, concluding that revascularization reduces the risk of major amputation or inpatient death for patients with CLI when compared to conservative therapy [19].

This study showed that 16 (72.73%) patients reported improvement in pain in Group A as compared to 17 (85%) patients in Group B. Thus, a higher percentage of patients in Group B reported relief from pain, but the result was not statistically significant (p = 0.554). Therefore, no significant difference was found in pain relief between conservative management and stenting in patients with PAD. However, it was observed that a slightly higher percentage of patients reported improvement in pain after angioplasty and stenting.

During the study period, four (18.18%) patients in Group A succumbed to the disease as compared to one (5%) patient in Group B. Although mortality was lower in the intervention group, the result was not statistically significant (p = 0.401). A similar result was observed in the COMPASS trial, which showed that when rivaroxaban 2.5 mg twice daily was combined with aspirin, it lowered the incidence of major adverse limb events and related complications, although the result was significant in the COMPASS trial [20].

In the domain of “Looking after myself,” a significantly higher proportion of patients in Group B reported no problems (100%) compared to 61.11% in Group A (p = 0.011), indicating better self-care ability in Group B. Thus, this study indicates that patients who underwent revascularization with angioplasty and stenting can take care of themselves and reduce their dependence on others.

This prospective study demonstrates that angioplasty with stenting offers a clear benefit in terms of wound healing, self-care, and health perception for infrainguinal PAD compared to medical management alone. Trends toward reduced amputation and mortality, although encouraging, were not statistically significant due to the sample size. The intervention group’s superior quality-of-life outcomes highlight the broader impact of revascularization beyond limb salvage.

This study has the limitation of a shorter observation period after the intervention. A longer observation time can provide the patency rate of the stent after intervention. A long follow-up period can also determine the time period for which medical management can be safely considered for patients with CLTI. In view of the better wound healing observed among the intervention group, this study provides scope to conduct this type of interventional study in a larger group of patients.

## Conclusions

This study found that angioplasty with thrombolysis and stenting significantly improves wound healing, self-care, and subjective health outcomes for patients with infrainguinal PAD. These results support the use of endovascular intervention as first-line therapy for Fontaine III-IV and Rutherford 4, 5, and 6 category patients. There is scope for further study of the effect of endovascular intervention in patients with PAD, which can significantly reduce limb loss and mortality and improve pain relief, wound healing, and quality of life.
